# Deep learning assisted holography microscopy for in-flow enumeration of tumor cells in blood†

**DOI:** 10.1039/d2ra07972k

**Published:** 2023-02-02

**Authors:** Anirudh Gangadhar, Hamed Sari-Sarraf, Siva A. Vanapalli

**Affiliations:** a Department of Chemical Engineering, Texas Tech University Lubbock TX 79409 USA Siva.Vanapalli@ttu.edu; b Department of Electrical and Computer Engineering, Texas Tech University Lubbock TX 79409 USA

## Abstract

Currently, detection of circulating tumor cells (CTCs) in cancer patient blood samples relies on immunostaining, which does not provide access to live CTCs, limiting the breadth of CTC-based applications. Here, we take the first steps to address this limitation, by demonstrating staining-free enumeration of tumor cells spiked into lysed blood samples using digital holographic microscopy (DHM), microfluidics and machine learning (ML). A 3D-printed module for laser assembly was developed to simplify the optical set up for holographic imaging of cells flowing through a sheath-based microfluidic device. Computational reconstruction of the holograms was performed to localize the cells in 3D and obtain the plane of best focus images to train deep learning models. We developed a custom-designed light-weight shallow Network dubbed s-Net and compared its performance against off-the-shelf CNN models including ResNet-50. The accuracy, sensitivity and specificity of the s-Net model was found to be higher than the off-the-shelf ML models. By applying an optimized decision threshold to mixed samples prepared *in silico*, the false positive rate was reduced from 1 × 10^−2^ to 2.77 × 10^−4^. Finally, the developed DHM-ML framework was successfully applied to enumerate spiked MCF-7 breast cancer cells and SkOV3 ovarian cancer cells from lysed blood samples containing white blood cells (WBCs) at concentrations typical of label-free enrichment techniques. We conclude by discussing the advances that need to be made to translate the DHM-ML approach to staining-free enumeration of actual CTCs in cancer patient blood samples.

## Abstract 2

Currently, detection of circulating tumor cells (CTCs) in cancer patient blood samples relies on immunostaining, which does not provide access to live CTCs, limiting the breadth of CTC-based applications.

## Graphical abstract

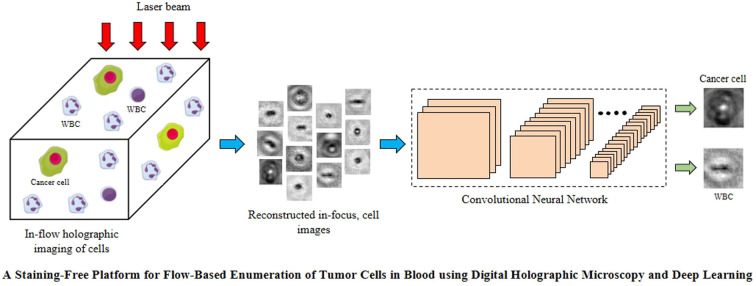

## 1. Introduction

Circulating tumor cells (CTCs) shed from primary tumors have been identified in the blood of cancer patients.^1^ Given their critical role in the metastatic cascade, these rare cells serve as novel clinical biomarkers to assess the tumor burden enabling effective monitoring of therapeutic treatment strategies.^2–4^ This has led to numerous efforts to develop methods to identify and characterize these information-laden cells. In comparison to traditional tissue biopsies, these blood-based “liquid biopsy” approaches are minimally invasive and can be carried out routinely enabling more frequent monitoring of CTCs.^5^

CTCs occur at extremely low counts in the blood (1 to >1000 CTCs per 10^9^ blood cells),^6–10^ motivating the development of methods to isolate them. Two main types of separation strategies are employed to isolate CTCs. In the affinity-based approach, specific antibodies are immobilized onto solid substrates, whereupon CTCs expressing the target antigen are selectively captured and recovered for subsequent downstream processing.^11–14^ Although affinity-based capture promises high specificity, this method suffers from marker-dependence, which limits capture of those CTCs which do not express the particular antigen,^15^ resulting in a lower yield of CTCs. In contrast, label-free methods take advantage of the differences in biophysical properties such as size,^16–18^ deformability,^19^ dielectric properties^20,21^ and density^22^ between CTCs and blood cells. The purity of the enriched sample with label-free techniques defined as the ratio of CTCs to nucleated white blood cells (WBCs) ranges from 1 : 100–1 : 1000 per mL after enrichment.^23–25^

The presence of background WBCs in the isolated sample makes it necessary to perform immunostaining – the current gold standard to identify CTCs using antibodies targeting CTC-specific antigens. However, the use of immunostaining limits the full translational utility of CTCs. For example, there is growing interest in *ex vivo* culture of CTCs for biomarker and drug discovery^26–28^ and the bottleneck here is to know which patient samples have sufficient CTCs for culture expansion since blood samples may not even contain CTCs or have very low counts depending on cancer type, stage and treatment course.^29,30^ Given that the immunostaining process kills CTCs, it becomes impossible to know apriori which patient samples are suitable for CTC culture. Therefore, it is useful to develop a staining-free quick screening tool that will enable rapid assessment of CTC load allowing informed decisions on the type of downstream assays that could be performed with live CTCs isolated from each cancer patient blood sample.

Staining-free approaches to detect CTCs could be potentially developed from bright-field images of CTCs and WBCs.^31^ However, marker-free CTC isolation methods (*e.g.* Vortex HT chip^32^ and Labyrinth chip^33^) often generate the rare cells in large sample volumes, requiring spinning down of the suspension to a localized spot on glass side to facilitate imaging, but this additional spin step can result in significant cell losses.^34–36^ Addressing this issue, previously, a study from our laboratory has shown that application of in-line digital holographic microscopy (DHM) to *in vitro* tumor cells flowing in a microchannel, can process large sample volumes and fingerprint individual cells,^37^ without the need to concentrate the sample. This flow-through DHM approach is attractive since 3D information of flowing objects is encoded onto a 2D image recorded on the camera sensor. The resulting interference patterns or holograms can then be computationally reconstructed to obtain 3D positional information, size and intensity features that can be used to differentiate cell types.^38,39^

Unlike our previous work, the technical advances we make in this study are: (i) we simplified the optical set up to develop a compact holographic module (cHol) that has a small foot-print and can be instrumented to a standard inverted microscope. This is in contrast to the complex and large foot-print optical set up used previously (ii) we incorporated a microfluidic sheath-flow device to eliminate wall-induced interference fringes and reduce the spread in cell velocities (iii) we implemented a convolutional neural network (CNN) on the in-focus images extracted from the holograms to train a machine learning (ML) model that can distinguish between cancer cells and WBCs and enumerate the former in flow. This is in contrast to previous work that used extracted features to distinguish *in vitro* cells and white blood cells. This feature engineering approach can be limiting as it requires apriori knowledge and features used for one type of cancer cells may not be applicable to other types of cancer cells.

Harnessing these technical advances, we focus on addressing the following questions using *in vitro* cancer cells as surrogate CTCs: (i) which deep learning models are suited to identifying cancer cells among WBCs in terms of accuracy, minimal false positives and inference times? (ii) How does the WBC load in the sample impact the limit of detection (LoD) of the ML model? (iii) Can the ML framework be generalized to different types of cancer cells? Addressing these questions is important before advancing the deep learning approach to the challenging problem of label-free detection of actual CTCs in patient blood. Thus, the focus of the present work is to advance the holographic hardware as well as deep learning tools to detect different types of tumor cells in a mixed population with WBCs that will lay the foundation for future studies with blood from cancer patients.

Our paper is organized as follows: we begin with a description of our cHol approach in Sec. 2.1, followed by detailing the analysis workflow developed for flow-based interrogation of tumor cells in Sec. 2.2. Next, in Sec. 2.3, we develop a customized, shallow convolutional neural network (CNN), demonstrating that its classification performance is not only comparable but also superior to significantly deeper models published in the literature. Sec. 2.4 focuses on implementing the decision thresholding method to significantly reduce the false positive rate of the selected model. This is tested on artificial mixed datasets prepared from the pure population images of both cell types. In Sec. 2.5, we apply our cHol-ML framework to enumerate breast cancer cells spiked into lysed blood at various target concentrations. Finally, in Sec. 3, we discuss the implications of our findings and the challenges that need to be addressed to translate our work to actual CTCs in cancer patient blood.

## 2. Results

### 2.1 Compact digital holographic microscopy (cHol) module

Digital holographic microscopy (DHM) has been widely used as a quantitative imaging tool in various cell-based applications such as morphology characterization using optical properties like intrinsic refractive index,^40,41^ mapping 3D trajectories in flow^42,43^ and label-free detection of diseases such as cancer.^44–46^ Two types of DHM configurations have been commonly utilized, in-line and off-axis, so named depending on whether the light source, sample and recording medium share the same optical axis or not.^42,47–49^ A common drawback is that these optical set ups can be complex, requiring precision alignment of multiple components, making it challenging to easily translate this powerful technique to laboratories.

**Fig. 1:**
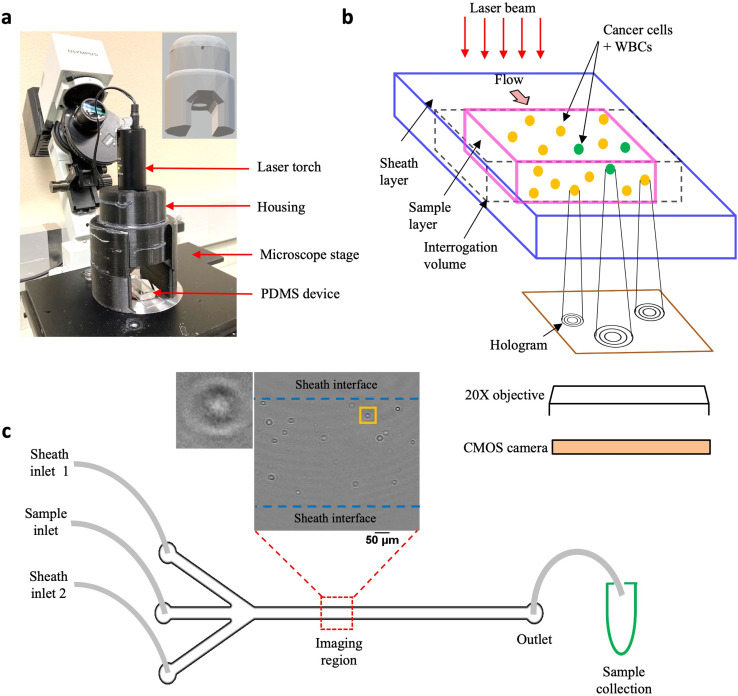
Experimental setup. (a) Compact holographic assembly. The laser torch is mounted on a movable housing. Inset shows the 3D design of the housing, the circular aperture allows the laser beam to pass through, illuminating the sample coherently; (b) schematic showing in-flow holograms of a mixed sample containing MCF-7 cells and WBCs imaged with a 20× microscope objective and recorded digitally, using a CMOS, high-speed camera; (c) PDMS-based sheathing device used for flowing the sample contains two inlet channels for entry of the sheath fluid (PBS) and a central inlet for sample injection. Imaging is performed in a region near the center of the channel far away from the entrance. Inset shows a representative cleaned hologram recorded using the cHol setup, scalebar is 100 μm. By imposing a sheath flow, the cells are confined to a region away from the channel walls. Post-DHM imaging, sample is collected from the device outlet for subsequent fluorescence imaging to obtain the ground truth counts.

Addressing this need, we have developed the cHol module that sits on the stage of a standard inverted microscope (Fig. 1a). It consists of two components: (1) a laser torch which contains within it a laser diode, a collimating lens and an automatic power control circuit and (2) a 3D-printed assembly to mount and position the torch. The emitted laser beam has a wavelength of 635 nm and a minimum diameter of 3 mm. The source-to-sample distance is about 45 mm while the maximum distance between the sample and the focal plane of the microscope objective is 0.53 mm.

The schematic in Fig. 1b illustrates the hologram recording process. Cells flowing through a microfluidic channel scatter the incident laser light. The scattered (object) wave interacts with the unobstructed (reference) wave to form interference fringes or holograms at the focal plane of the microscope objective. These are subsequently recorded digitally on a CMOS sensor using a high-speed camera. Since the source, sample and detector are located along the same optical axis, holographic imaging is performed using the in-line configuration. In the in-line mode, reconstructed intensity fields are obtained from the corresponding amplitudes while the phase information gets corrupted due to the twin-image artefact, requiring additional recovery strategies.^50–52^ We performed static localization studies^48^ to optimize the objective magnification, plane-to-plane spacing for hologram reconstruction, and recording distance defined as the distance between the microchannel floor and the focal plane of the microscope objective. We found that a recording distance of 200 μm, with a 20× magnification objective was optimal for acquiring holograms, and reconstruction was performed with a spacing of 5 μm (Fig. S1a†).

Unlike, our previous study, here we modified the microchannel geometry to include a trifurcated inlet (Fig. 1c). The microchannel had a width (*W*) and height (*H*) of *W* × *H* = 800 μm × 330 μm respectively. The trifurcated inlet was used to introduce a sheathing flow to overcome two main issues: (1) undesirable interference fringes from channel sidewalls and (2) cells close to the wall move very slowly, creating multiple instances in the hologram recordings. These multiple cell counts could introduce large errors especially for the near-wall cells. By optimizing the flow rate of the sheath fluid relative to the sample flow rate, the cell suspension was confined to central width of ≈500 μm, and *a* ≈ 150 μm of sheath fluid on either side (Fig. S1b and c†). At an optical resolution of 1 μm per pixel, a single hologram corresponds to an image volume of 500 μm × 800 μm × 330 μm ≈ 0.13 μL of sample. To remove multiple cell counts, a frame rate of 420 fps was chosen such that the fastest moving cell only appeared in a single frame. Typically, it was observed that cells appeared in a maximum of 2–3 sequential frames. These cells were mostly located near the sample–sheath interface wherein, the parabolic fluid velocity profile caused them to move much slower as compared to cells near the channel centerline. At the selected frame rate, 10 100 holograms were captured so that tumor cells could be enumerated from 1 mL of sample volume flowing through the microchannel.

### 2.2 Basic workflow of DHM-ML detection of cancer cells

To implement the DHM-ML strategy, we initially used MCF-7 breast cancer cells as a surrogate for CTCs recognizing that some characteristics of patient-derived CTCs could be different from that of MCF-7 cells. Blood was processed using lysis buffer to eliminate red blood cells and centrifuged to isolate WBCs. Holograms were obtained of pure populations of MCF-7s as well as WBCs that were used for selection of ML models. Mixed samples containing MCF-7s spiked into WBCs were used to test the performance of the optimal ML model. The MCF-7 cells were labeled with a fluorescent marker to establish ground-truth validation of counts obtained from the DHM-ML strategy.

**Fig. 2:**
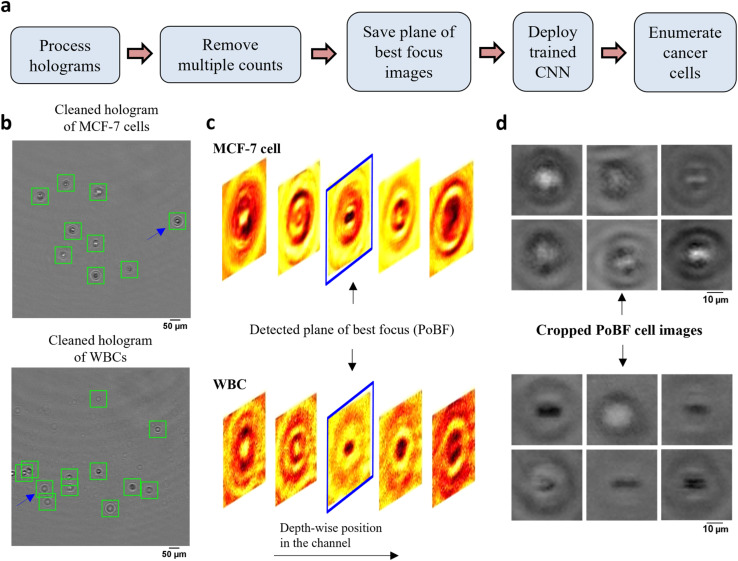
DHM-ML workflow including extraction of plane of best focus images of cells. (a) DHM-ML analysis workflow; (b) cells detected and localized as shown by the green squares are overlaid on the cleaned holograms of pure cell populations; (c) full volume numerical reconstruction allows 3D localization of cells, enabling detection of the plane of best focus (PoBF). Reconstructed images of the cell highlighted by the blue arrow in (a) are shown at various axial distances. The image highlighted in blue shows the detected plane of best focus (PoBF); (d) examples of cropped, 36 × 36 PoBF images are shown for pure MCF-7 cells (top) as well as WBCs (bottom).

The computational analysis workflow for the DHM-ML strategy is shown in Fig. 2a. To profile every cell in the field of view (FOV), the acquired raw holograms are first denoised by subtracting each raw hologram with an object-free background, estimated as the average of 10 100 raw holograms. Subsequently, numerical reconstruction is performed on the resultant hologram to localize in 3D all the cells in the image volume. From the reconstructed image stack, the 2D center of each cell is identified initially which enables its detection in the denoised hologram (Fig. 2b). Equipped with this information, axial intensity maps are generated for each cell which allows us to obtain the plane of best focus (PoBF) or in-focus image (Fig. 2c). Examples of PoBF images for MCF-7s and WBCs are shown in Fig. 2d. Our methodology for background subtraction, reconstruction of holograms and PoBF selection has been described in detail previously.^37,48^

Next, multiple counts of cells are removed since the flow is laminar where the motion of cells is predominantly in the streamwise direction and the cross-stream (*y*) and axial (*z*) velocity components can be neglected. This allows us to use their detected *y*, *z* positions to remove multiple counts. Cells were identified and eliminated if the following conditions were met: |*y*_q,*i*+1_ − *y*_p,*i*_| ≤ 3 μm and |*z*_q,*i*+1_ − *z*_p,*i*_| ≤ 50 μm. These criteria were verified by manually tracking the detected *y*, *z* positions of about 100 MCF-7 cells as well as WBCs selected at random. Interestingly, we found appreciable changes in the axial positions of cancer cells between two successive frames, consequently, a relatively lenient *z* criterion is applied.

After removing the multiple counts, the PoBF images (Fig. 2d) are cropped to 36 × 36 pixel sub-images using the centroid location of the cells. These sub-images are fed to the trained deep learning model which classifies them as MCF-7 or WBC. Applying an optimized decision gating on the predictions, we obtain enumerated counts of MCF-7 cells in the analyzed sample. It is worth noting that the entire computational analysis pipeline is automated and does not require any supervision.

### 2.3 Development of custom ML model and its comparison with existing deep learning models

In this section, we develop a light-weight custom CNN architecture and evaluate its performance against published DL models in classifying MCF-7 breast cancer cells from WBCs using holographic reconstructed in-focus images. To achieve this, we initially tested two CNN architectures: (1) ResNet-50, a popular choice for image-based classification tasks^31^ and (2) a custom-built, shallow network, s-Net, with significantly fewer parameters. Advantages of using the latter mainly include shorter training and testing times without the need for high computational power. We evaluated the classification performance of the s-Net and compared it with that of ResNet-50.

**Fig. 3:**
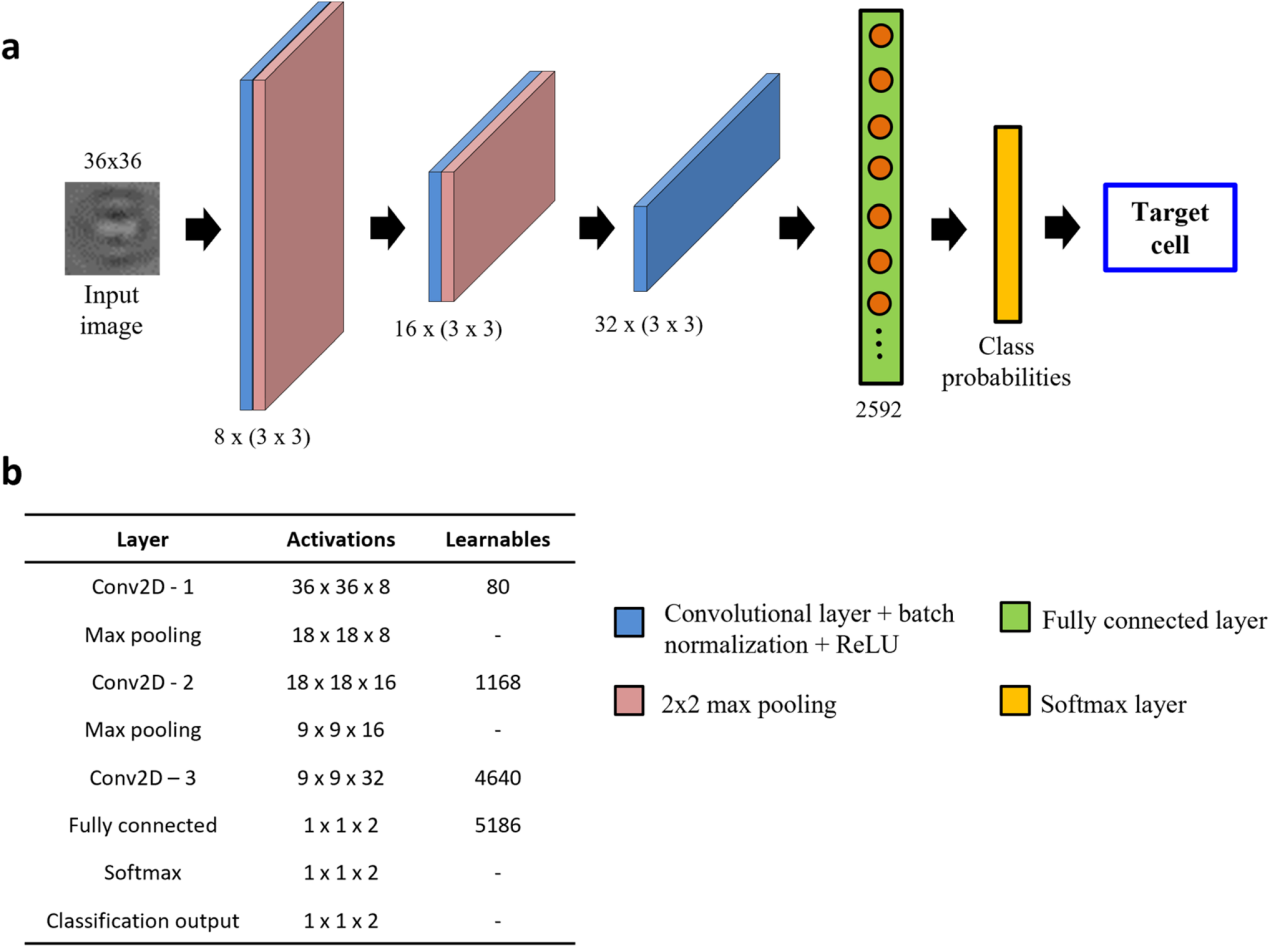
Architecture of custom-built s-Net. (a) Network architecture consisting of 8 layers: 3 convolutional, 2 max pooling, 1 fully connected, Softmax and output classification; (b) table showing the number of activations and learnables (sum of weights and biases) in each layer. The Network learns parameters only in the convolutional and fully connected (FC) layers.

The s-Net architecture is shown schematically in Fig. 3a. A 36 × 36 PoBF cell image is fed to the network as input. Three convolutional layers make up the backbone with a filter size of 3 × 3 and a stride of 1. The number of filters increases from 8 to 32 and “same” padding is used to ensure that image size is conserved before and after the convolution. This is followed by batch normalization and ReLU activation. The output image is passed to a max pooling layer where it is down sampled using a window size and stride of 2. The third convolutional layer is succeeded by a flattened fully connected (FC) layer containing 2592 nodes. Finally, a Softmax layer produces the class (MCF-7, WBC) probabilities which result in a prediction. The activations and number of learnable parameters (weights + biases) are tabulated in Fig. 3b.

For model evaluation, training and testing set images were generated from pure population experiments, *i.e.*, only MCF-7 or WBC images. Cancer cells were not fluorescently tagged for this task. A total of 52 340 images were used per class. A 70/30 split was used to randomly generate the training and testing datasets which yielded 36 638 and 15 702 class images respectively. We used a minibatch size of 32 and the number of epochs was set at 20. To compute the network weights, Adam optimizer with an initial learning rate of 10^−3^ was selected. Model optimization yielded the same three hyperparameter values for both models. It is worth noting that our shallower s-Net model (∼11 000 learnable parameters) yielded a total training time of 15 hours while training the considerably deeper ResNet-50 (>25 million learnable parameters) took almost 3 days with our GPU (NVIDIA GeForce GTX 1060, 6 GB)-enabled system (32-Core Processor, 3.00 GHz, 32 GB RAM).

**Fig. 4:**
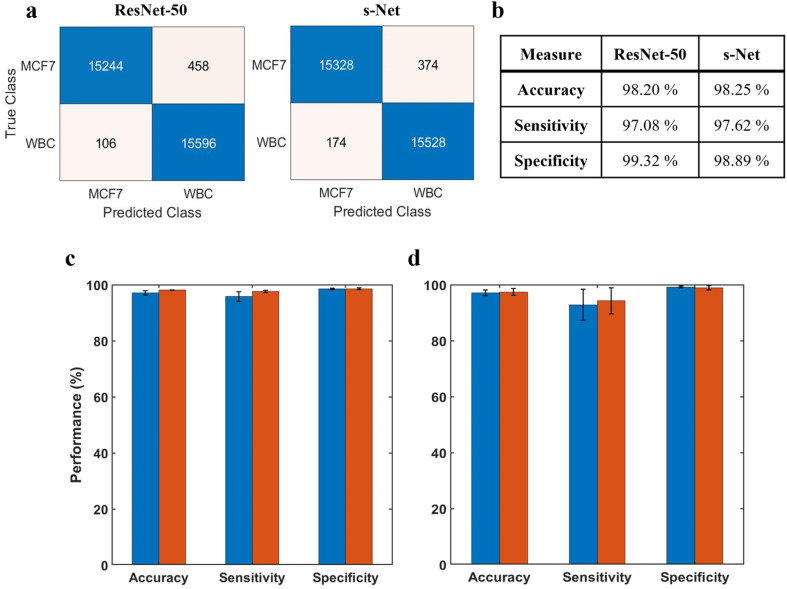
Evaluation of ML model performance. (a) Confusion matrices showing number of TPs, TNs, FPs and FNs for both ResNet-50 and s-Net from a representative trial (b) ML performance is evaluated by determining the accuracy, sensitivity and specificity computed on the test dataset. Results are shown for both network architectures tested (c) repeatability of the trained CNN. The network is serially trained multiple times with the same dataset; error bars indicate the standard deviation of five trials; (d) inter-trial variability of the CNN. The trained CNN is tested on multiple datasets; error bars indicate the standard deviation of three independent trials. In (c) and (d), blue corresponds to ResNet-50 and red corresponds to s-Net.

Before discussing the CNN performance, it is important to define these measures along with some ML-specific terminologies: True positives (TPs) refer to MCF-7 cells classified correctly; True negatives (TNs) refer to WBCs classified correctly; False positives (FPs) refer to WBCs incorrectly classified as MCF-7 cells; False negatives (FNs) refer to MCF-7 cells incorrectly classified as WBCs. In terms of performance measures, we define accuracy A = (TP + TN)/(TP + TN + FP + FN); sensitivity Se = TP/(TP + FN) also known as true positive rate (TPR); specificity Sp = TN/(TN + FP) also known as true negative rate (TNR); and false positive rate (FPR): FPR = FP/(FP + TN). Fig. 4a shows the confusion matrices computed on the testing dataset for both networks. Performance of the trained networks was evaluated by computing the accuracy, sensitivity and specificity on the testing dataset (Fig. 4b). Overall, we find that both networks perform reasonably well.

The ML training process is generally subjected to a certain degree of uncertainty introduced by the random initialization of learnable network weights. This could lead to variations in the classification performance that needs to be accounted. To evaluate model repeatability, the model was trained and tested with the same dataset multiple times. Results are reported in Fig. 4c, where the height of the bar indicates the mean value of 5 unique trials. From the standard deviations, we infer that the variability in network performance is inconsequential. Additionally, we evaluated the robustness of the trained model by testing it with datasets generated from 3 different trials (Fig. 4d). In this case, the model was trained only once prior to testing. Again, we observe very low variability between the trials.

It is evident that the performance of the s-Net is on par with the deeper, over parametrized ResNet-50 network. This comparable performance, in conjunction with its shorter inference time (24 seconds per 50 000 input images) in comparison with ResNet-50 (196 seconds per 50 000 input images) make it a suitable candidate to be used for the enumeration task. Besides ResNet-50, the performance of s-Net was also compared to state-of-the-art, lightweight CNN Networks such as LeNet-5 (ref. 53) and MobileNet^54^ (see Table S1,† where a smaller training set of 3500 examples for each class, and a testing set of 1500 examples was used for comparing performance). Evaluating classification accuracy, sensitivity and specificity, we found the performance of s-Net to be superior. It is interesting to note that among the four networks tested, s-Net has the least number of learnable parameters (*i.e.* 11 000 in s-Net *versus* 25.5 million in ResNet-50), highlighting the utility of shallow CNN networks for the classification task at hand. Motivated by these results, we use s-Net in subsequent studies.

### 2.4 Detection of tumor cells from artificially mixed datasets using decision thresholding

**Fig. 5:**
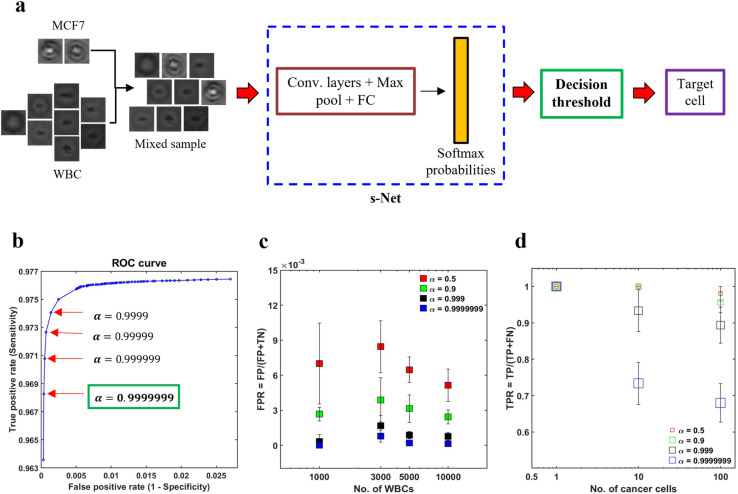
Reducing the false positives (FPs) using decision thresholding. (a) Schematic illustrating the application of decision thresholding for cancer cell classification; (b) selection of optimal threshold using receiver operator characteristic (ROC) analysis. A stringent threshold (*α*) of 0.9999999 is selected as it yielded the minimum FPR without any further change; (c) to demonstrate the applicability of the selected threshold in reducing the FPR, four artificially mixed samples were generated *in silico*. In these datasets, the number of MCF-7 cancer cells (*N*_MCF−7_) was kept constant at 100, while the number of WBCs (*N*_WBC_) was varied from 1000–10 000. It was ensured that no overlap between the two cell types existed across the four datasets. For increasing WBC counts, the FPR is plotted for 4 values of *α*: 0.5 (red), 0.9 (green), 0.999 (black) and 0.9999999 (blue). Error bars indicate the standard deviation of three trials (*n* = 3); (d) effect of *α* on the TPR is shown. In these trials, three datasets were generated, wherein, *N*_WBC_ was fixed at 5000 and *N*_MCF−7_ was varied from 1–100. Error bars indicate the standard deviation of three trials (*n* = 3).

In spite of the s-Net's encouraging performance, its application to spiked samples is hindered by the presence of excessive false positives. By calculating the mean from five trials, we find that the FPR is 0.014, which indicates that the s-Net misclassifies approximately 14 WBCs out of 1000 as MCF-7 cells. This is deemed to be too high for our task and can result in a significant overestimation of target cell counts. In this section, we discuss decision gating, a strategy to reduce the false positive rate associated with classification.^55^ This method involves the application of a strict threshold on the output probabilities generated by the ML Network in order to classify cells. The schematic in Fig. 5a gives an overview of the process. First, mixed population datasets are generated *in silico* from pure population PoBF images of untagged MCF-7 cells and WBCs. For every image fed to the s-Net in a serial fashion, the Softmax layer outputs class probabilities: *P*_MCF7_ and *P*_WBC_. A cell is classified as MCF-7 if *P*_MCF−7_ > 0.5. To decrease the FPR, it is necessary to use a decision threshold *α* greater than 0.5.

To determine the optimal value of *α* we conducted receiver operator characteristic (ROC) analysis, where model sensitivity (Se) and specificity (Sp) are computed on the testing set for different values of *α*. The trained s-Net and test dataset used is the same as that discussed in the previous section. The ROC curve shown in Fig. 5b shows the effect of varying the decision threshold on the true positive and false positive rates. Generally, an increase in decision threshold value *α* results in a reduction of both TPR and FPR. The lowest FPR was obtained at *α* = 0.9999999 which was selected as the optimal threshold. Higher values did not result in any further change in FPR.

The next step is to test the applicability of the selected decision threshold by testing it on artificially mixed datasets containing MCF-7 cells and WBCs. First, we sought to evaluate the effect of *α* on the FPR. In order to do this, four independent datasets were created where the number of MCF-7 cells (*N*_MCF−7_) was fixed at 100 but the number of WBCs (*N*_WBC_) was made to range from 1000–10 000. The rationale for doing so was that the FPR depends solely on the classification statistics computed on the negative class, *i.e.*, WBCs in our case. For this reason, *N*_MCF−7_ was kept constant. For each WBC count tested, we plot the FPR at four different decision thresholds. As shown in Fig. 5c, increasing the decision thresholding helps to significantly reduce the false positive rate. At the selected threshold, *i.e.*, *α* = 0.9999999, the mean FPR is measured to be 2.77 × 10^−4^. Additionally, contrary to the case of low *α*, where FPR is much more sensitive to the number of WBCs needed to be screened in the sample, at the selected threshold we find that the FPR is not varying significantly with WBC counts. This invariance in FPR suggests that as the number of background WBCs in the sample (*N*_WBC_) increases, the number of false positives also increase.

While the thresholding strategy has been effective in lowering the FPR, imposing such a strict boundary invariably lowers the number of true positives. This makes it important to evaluate the extent of decrease in TPR and assess whether this is acceptable or not. To achieve this objective, we created three mixed datasets where only the number of cancer cells were varied: *N*_MCF−7_ = 1, 10, 100 while the number of WBCs was kept constant (*N*_WBC_ = 5000). We find that TPR depends on both the number of MCF-7s as well as *α* (Fig. 5d). Evaluating at *α* = 0.9999999, we find that TPR decreased to about 0.7 for *N*_MCF−7_ = 10 and 100 respectively. In the dataset with only a single cancer cell, our s-Net model is able to detect it robustly at the selected threshold.

Overall, our analysis suggests that both the FPs and TPs depend on the decision threshold as well as counts of WBCs and MCF-7s. This highlights the potential limitation of the deep-learning model for label-free CTC enumeration. CTCs detected as WBCs would result in underestimating the tumor burden which could prove to be fatal for the cancer patient. On the other hand, scoring WBCs as CTCs would result in overpredicting the actual CTC counts, with the possibility of subjecting patients to unnecessary treatment regimens. Acknowledging this limitation, we evaluated how well the DHM-ML approach would characterize varying loads of MCF-7s in actual blood samples, which we discuss in the next section.

### 2.5 DHM-ML-assisted enumeration of tumor cells spiked into lysed blood

We applied the decision thresholding method to enumerate tumor cells from actual mixed samples containing a background of WBCs. Briefly, fluorescently labelled MCF-7 breast cancer cells were spiked at different target concentrations: 0, 10, 100 and 1000 mL^−1^. The concentration of WBCs was kept constant at either 1000 or 5000 cells per mL – this concentration is within the range expected from label-free enrichment techniques. To achieve flow-based interrogation of tumor cells, 10 100 holograms of mixed samples containing MCF-7 cells and WBCs were captured to image a total sample volume of 1 mL. Automated ML-based enumeration was carried out according to the earlier-mentioned procedure (see Fig. 2a). For validating the detected concentrations, we do not rely on the theoretical target counts but obtain separate ground truth counts. This was done by collecting the entire DHM-imaged sample from the outlet of the sheath device (∼1.4 mL total volume from both sample and sheath fluid) and performing fluorescence imaging to obtain the actual counts of MCF-7 cells (details in Sec. 5.7). Establishing a robust validation strategy was important since sample handling can introduce variability and serial dilution could lead to high errors at extremely low spiked concentrations.

**Fig. 6:**
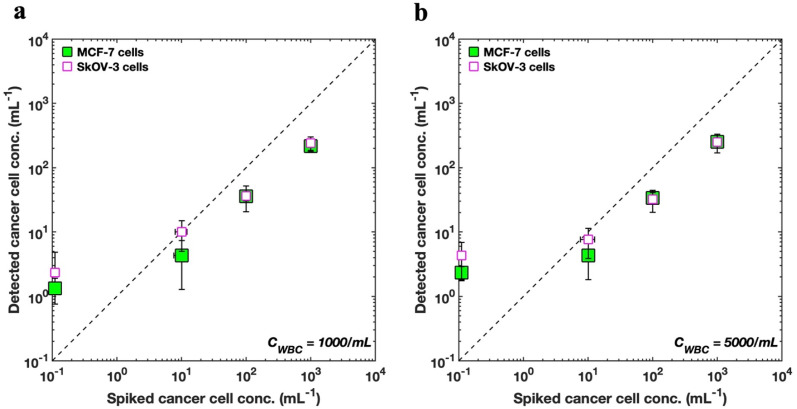
Comparing enumeration predictions for MCF-7 and SkOV3 cells. (a) Enumeration of SkOV3 cells into lysed blood using the retrained s-Net is compared with that of MCF-7 breast cancer cells using the original s-Net. WBC concentration is set as 1000 cells per mL; (b) comparison of enumeration results for the two cell types. WBC concentration is 5000 cells/mL. In both (a) and (b), horizontal error bars indicate the variability associated with the ground truth fluorescence counts, and many of them are smaller than the symbol.

Fig. 6 shows the enumeration results of spiked MCF-7 cells as predicted by the s-Net model with the data plotted on a log–log scale. To improve the accuracy of the recovered target cells, the optimized decision threshold *α* = 0.9999999 was applied. We find that the enumerated MCF-7 concentrations increase with increasing spiked cell concentrations, although the actual numbers do not quantitatively agree (see ESI Tables S2 and S3†). Thus, the DHM-ML approach can sufficiently distinguish between varying loads of cancer cells at high WBC background counts indicating that it can be considered as a qualitative screening tool to identify blood samples that have high number of CTCs.

We also evaluated whether the s-Net model could be used to detect other types of cancer cells. From a clinical perspective, this is important because it may lead to marker-free detection of CTCs from different cancers, without the need to identify reliable biological targets, which can be a major challenge with current labeled approaches. Therefore, we apply our existing s-Net model to enumerate *in vitro* SkOV3 ovarian cancer cells. To accomplish this, we retrained the s-Net model with the same architecture as before on the new SkOV3 cancer cells. The retrained s-Net model was applied to enumerate SkOV3 cells spiked at target concentrations of 0, 0, 100 and 1000 cells per mL into lysed blood. Detected concentrations are reported as the number of target (SkOV3) cells on 1 mL of actual sample analyzed. As before, ground truth data (Tables S4 and S5†) from the DHM-imaged sample collected downstream was also generated. Fig. 6 compares our results for detecting the spiked SkOV3 cells using the retrained s-Net with that of MCF-7 cells. At both WBC background concentrations tested, we generally observe good agreement in the predictions for both types of cancer cells. These results are encouraging since a generalized performance could be achieved using a shallow Network, without the need for off-the-shelf deep sophisticated DL networks.

As part of establishing the screening or enumeration potential of an assay, it is critical to report the limit of detection (LoD). This is defined as the lowest target cell concentration that can be reliably detected by an assay and is given by: LoD = *μ*_NC_ + 2*σ*_NC_.^56^ Here, *μ*_NC_, *σ*_NC_ denote mean and standard deviation of the negative control results, *i.e.*, the sample does not contain any analyte or cancer cells. To calculate the LoD for our system, we performed 3 negative control trials, where the samples contained WBCs but no tumor cells (Fig. 6). For MCF-7 cells, the LoD was 2.49 (*μ*_NC_ = 1.33, *σ*_NC_ = 0.58), and 3.49 cells per mL (*μ*_NC_ = 2.33, *σ*_NC_ = 0.58), for WBC concentrations of 1000 and 5000 cells/mL respectively. Likewise, for the SkOV3 cells, the LoD was 3 (*μ*_NC_ = 1, *σ*_NC_ = 1), and 4.33 cells per mL (*μ*_NC_ = 2.33, *σ*_NC_ = 1), for WBC concentrations of 1000 and 5000 cells per mL respectively. These results are comparable to the LoD reported for immunostaining where studies report antibody-based CTC counts of <2 per mL of blood in healthy donors.^32,33,57^

## 3. Discussion

Immunostaining is the current gold-standard for identifying CTCs from a background of blood cells. Despite being highly selective, this approach is time-consuming requiring multiple processing steps, and is dependent on the quality of antibodies. Importantly, this approach limits some critical CTC applications like *ex vivo* culture expansion of CTCs, and omic analysis where there are opposing needs to access live CTCs but also in selecting patients with adequate CTC counts. This gap can be addressed by developing staining-free approaches to detect CTCs. In this work we present the first steps to tackle this problem using deep learning models and improved holography hardware system. Our results show that the DHM-ML strategy is promising, but additional research efforts are needed to translate this approach to blood samples of cancer patients, which we discuss below.

### 3.1 DHM-ML approach can be integrated with a variety of upstream enrichment techniques

In our DHM-ML study we used a sheath device in which a mixture of MCF-7 cells and WBCs were evaluated. Many CTC isolation technologies enrich the CTC population using mechanical filters,^58,59^ inertial focusing^8,25,60,61^ and negative selection.^62–64^ Our approach is compatible with any of these approaches since the enriched samples can be collected and introduced into the sheath device for enumeration. This a major advantage of our DHM-ML strategy as it allows broader application to a variety of CTC-enrichment technologies.

Our results in Sec. 2.4 show that when the number of background WBCs to be screened is high, the ML model needs to balance between FPR and TPR, but at low WBC counts it can perform with greater accuracy. Specifically, for *N*_WBC_ = 1000, our ML model predicts 0 false positives (for *α* = 0.9999999) which is quite promising. Testing this experimentally, we performed the analysis for negative control samples at low (1000, 5000 per mL) WBC loads. Particularly, when the sample contained 1000 WBCs per mL, the false positive count was a mere 1.33 which is in good agreement with our result from *in silico* trials.

Given the limitation of the deep-learning approach for high WBC counts, it is important to apply enrichment strategies to reduce the background WBC counts, as this will lead to lower FPs. Different CTC isolation technologies report different levels of sample purity after enrichment, *i.e*., producing different background WBC counts. For example, label-free technologies such as the Vortex HT chip^32^ and Labyrinth^33^ produce background WBC counts in the range of 25–1000 per mL sample. At such high levels of purity, we anticipate that the LoD can be lowered and the FPR reduced even further. Therefore, implementing high-fidelity enrichment strategies prior to DHM-ML detection should bring a major advance in staining-free CTC enumeration.

### 3.2 Expanding cHol-ML approach to patient samples requires dedicated computational hardware

Access to live CTCs is the next frontier in liquid-biopsy research. However, significant intra-patient and inter-patient variability exists in CTC counts.^65^ Some of the blood samples may not even contain CTCs or may have very low counts bringing ambiguity into the type of downstream assays that could be pursued. Therefore, there is a need for a quick screening tool that can assess live CTCs and our DHM-ML technique presents a promising avenue to address this gap. Given that CTCs are rare, high volume of blood needs to be processed which may increase the computational burden on hologram processing. However, implementing an enrichment step eliminates the need to computationally enumerate blood cells. As an example, the Labyrinth chip is able to process 5 mL of patient whole blood and deliver a 2 mL CTC-enriched sample volume. Using our cHol setup, this sample can be flowed through our sheath channel and the entire sample can be imaged by recording 21 000 experimental holograms (10 100 holograms for screening 1 mL of sample). It takes ∼5 seconds to process a single hologram on a standard desktop computer with 4 CPU cores. At this rate, we estimate that the entire sample can be analyzed, and tumor cell counts enumerated from 5 mL whole blood in ∼29 hours. This computational time can be reduced by integrating dedicated hardware such as Tensor core-enabled GPUs^66^ or increasing the number of CPU cores^67^ for parallelized operations to fast-track our analysis.

### 3.3 Application of DHM-ML to cancer patient blood samples

In our work, we used MCF-7 and SkOV3 cells as a proxy for CTCs, which is a limitation for translating our results to patient-derived CTCs. The challenge with developing ML models with real CTCs is generation of ground-truth data. Fluorescent markers capable of tagging live CTCs, coupled with additional morphological features (*e.g.*, cell size, nucleus-to-cytoplasm ratio) could be used to label CTCs for ML model development. A few studies are emerging addressing this gap. For example, Wang *et al.*^31^ used a Carbonic Anhydrase 9 antibody in conjunction with Calcein AM to differentiate CTCs from blood cells. In another study by Shao *et al.*,^68^ CTCs from prostate cancer patients were successfully labeled using a class of near-infrared Heptamethine Carbocyanine dyes. Although these advances are promising, further work is needed to develop robust markers capable of labeling CTCs across different types of cancers.

Another major hurdle for ML model development is that since these CTCs occur at extremely low frequencies (1 to >1000 cells per mL) in blood, collecting an adequate amount of ground truth data would require analysis of many blood specimens. Collecting large number of blood samples can be a logistical challenge. There are two main approaches to deal with this challenge: (1) recently, DL-based approaches such as Generative Adversarial Networks (GANs) have demonstrated sufficiently good performance with small or unbalanced datasets and can be explored; (2) for generating larger training datasets, a combination of patient-derived CTCs and *in vitro* cell lines may be used for the positive class.^31^

## 4. Conclusions

CTCs have extensive applications in cancer research, with emerging needs focusing on access to live CTCs for ex vivo culture and drug testing. Immunostaining, which is the standard procedure for identifying whether CTCs are present in a given patient sample, precludes access to live CTCs. Given that apriori knowledge of which patient samples have sufficient CTCs is required for sample selection, there is a need for staining-free techniques that can enumerate CTCs. Such label-free enumeration techniques, even if they are not perfect, can be very useful to select patient samples that are conducive to downstream applications such as *ex vivo* culture and drug testing. In this study, we employ digital holography microscopy, microfluidics and deep learning to tackle the first steps in addressing this challenge.

The use of deep learning neural networks for identification of cancer cells among blood cells is still in its infancy with some studies reporting results on static specimens.^69–72^ Building on these studies, here we explore whether flowing cancer cells in a microfluidic sheath device can be detected among a background of WBCs, by combining digital holography microscopy and deep learning models. To our knowledge, such an approach has not been evaluated and here we work with *in vitro* tumor cells to optimize and assess the performance of our approach.

We make significant technical advances including (i) introducing a compact holographic module which, unlike traditional setups, does not require precision alignment of multiple optical components and can be easily adapted across research laboratories and clinical settings enabling its widespread utility (ii) a microfluidic sheath flow device that eliminates near-wall fringes and improves accuracy of cell enumeration (iii) development of a custom-built s-Net model that is lightweight, fast and has superior performance than existing deep learning models (iv) successful demonstration of the DHM-ML approach to detect breast and ovarian cancer cells with limits of detection that are comparable to standard immunostaining methods.

In summary, our DHM-ML strategy represents the first step in developing CNN-based deep learning-based frameworks for label-free enumeration of tumor cells among a background of WBCs. While transitioning to patient samples still requires advances, the approach presented holds promise potentially lending to a quick screening tool for label-free enumerations of CTCs.

## 5. Materials and methods

### 5.1 Cell culture and procurement

A stock of MCF-7 breast cancer cells was obtained from American Type Cell Collection (ATCC, Manassas, VA). The cells were cultured using Dulbecco's modified Eagle medium (DMEM, Gibco, Gaithersburg, MD) supplemented with 10% Fetal Bovine Serum (FBS, Gibco), 1% Penicillin/Streptomycin (Gibco) and 1% sodium pyruvate (Gibco). They were incubated at 37 °C in a humidified atmosphere containing 5% CO_2_ to maintain ambient conditions. Ready to use SkOV3 cells in suspension were procured from Dr Wei Li's laboratory (Chemical engineering, Texas Tech University).

### 5.2 Fluorescent labeling of tumor cells

MCF-7 cells were fluorescently tagged with CellTracker™ Green CMTPX dye (Invitrogen™, Waltham, MA). A stock solution of 10 mM was prepared according to manufacturer's instructions. From this, a 10 μM working solution was prepared by diluting the stock in serum-free media. This solution was then added to the adherent cells in the tissue culture flask followed by an incubation step at 37 °C for 45 minutes. Subsequently, the cells were washed thrice with 1× phosphate buffered saline (PBS, Gibco) to remove any excess dye. The washed cells were trypsinized and resuspended in 500 μL of filtered, fresh media. SkOV3 cells in suspension were tagged according to the manufacturer's instructions.

### 5.3 Isolation of WBCs

Fresh human whole blood was purchased from BioIVT (Westbury, NY). 10 mL of blood was drawn from healthy donors into vacutainer tubes containing K2EDTA as anticoagulant and shipped on the same day. WBCs were isolated using ACK lysis buffer (Quality Biological, Gaithersburg, MD). First, 1 mL whole blood was incubated with 10 mL lysis buffer at room temperature for 5 minutes. This was followed by centrifugation at 2000 rpm for 5 minutes. After discarding the supernatant, the pellet was resuspended and mixed gently in 5 mL of lysis buffer following which the incubation and spinning steps were repeated. Subsequently, the supernatant was extracted and care was taken to ensure that the red RBC pellet was removed without disturbing the yellowish-white WBC pellet that was finally resuspended in 1× PBS.

### 5.4 Sample preparation

Cancer cell and WBC stock suspensions were passed through a 30 μm filter (CellTrics, Norderstedt, Germany) to remove any debris or other impurities. Cell concentrations were measured using a Neubauer hemocytometer. Cancer cells were counted three times and the average was recorded as the stock concentration. Pure population samples contained a concentration of 100 000 cells per mL. Mixed samples containing MCF-7/SkOV3 cells at spiked concentrations of 10, 100 and 1000 mL^−1^ and WBCs (1000, 5000 and 90 000 per mL) were prepared in 1× PBS.

### 5.5 Microfabrication

The sheath microchannel, 800 μm wide and 330 μm deep was fabricated using soft lithography.^73^ Negative photomasks were first designed in AUTOCAD (v. 2019, Autodesk, San Rafael, CA) and printed. SU-8 2050 (MicroChem Corporation, Westborough, MA), a negative photoresist, was used to prepare the mold. To achieve the large depth, a 2-step spin coating procedure was followed. At each step, the target height was selected to be 170 μm. The wafer with the etched pattern underwent trichloro (1H, 1H, 2H, 2*H*-perfluorooctyl) silane (Sigma Aldrich, St. Louis, MO) treatment for 24 hours. To prepare the devices, PDMS pre-polymer and the curing agent (Sylgard 184 Silicone Elastomer kit, Dow Corning Inc., Midland, MI) were mixed in a 10 : 1 *w*/*w* ratio, degassed, poured on the mold and cured in a 70 °C oven for 2 hours. The cured PDMS replicas were cut and peeled. Inlet and outlet fluidic ports were made using a 1 mm hole puncher (Instech Laboratories, Plymouth Meeting, PA). This was followed by plasma treatment (Harrick Plasma, Ithaca, NY) for 90 seconds to irreversibly bond the PDMS cutout to a 25 mm × 75 mm × 1 mm glass slide (Thermo Fisher, Waltham, MA). The hydrophilic devices were then incubated at 70 °C for 15 minutes to strengthen the surface bonding. Prior to experiments, 1 mL of 1% (w/v) Pluronic® F-127 (Sigma Aldrich, St. Louis, MO) prepared in 1× PBS was flowed through the microchannel at 100 μL min^−1^.^33^ This was done to prevent cell adhesion to the PDMS channel walls.

### 5.6 Digital holographic microscopy (DHM) imaging

The holographic setup consists of a laser torch (LDM 635, Thorlabs, NJ, USA) placed in a 3D-printed housing. The emitted laser beam (*λ* = 635 nm, 4 mW, diameter: 3 mm × 5 mm) serves as a coherent light source and is operated in the continuous wave (CW) mode. The imaging field of view (FOV) is 800 × 800 (in pixels) and the depth of field (or channel depth) is 330 μm. The flow is actuated by a syringe pump (PHD 2000, Harvard Apparatus). The flow rates of the sample and sheath fluid streams are 2.5 and 0.5 mL min^−1^ respectively, resulting in a total flow rate of 3.5 mL min^−1^. A frame rate of 420 frames per seconds is used in our experiments. The holograms are magnified by a 20× (1 μm per pix.) objective (20×, NA = 0.45, Olympus) with the hologram plane located 200 μm below the microchannel floor. Subsequently, they are recorded on a CMOS sensor of a high-speed camera (Phantom v310, Vision Research), facilitated by the PCC software (Phantom, Vision Research). An exposure time of 35 μs is used. 10 100 raw holograms are captured in order to image 1 mL of sample volume which takes about 24 seconds at the imposed frame rate.

### 5.7 Post-DHM fluorescence imaging for ground truth count generation

The DHM-imaged sample was collected and transferred to a 96-well plate (Greiner Bio-One, Monroe, NC), with each well containing 150 μL of sample volume. After allowing the cells to settle for about 20 minutes, imaging was performed using an Olympus IX81 epifluorescence microscope (Massachusetts, USA). By using a programmable stage (Thorlabs, New Jersey, USA), images were recorded in an automated fashion using the Slidebook 6.1 software (3i Intelligent Imaging Innovations Inc., Denver, USA). A digital monochrome camera (Hamamatsu, ImagEM X2 EM-CCD, New Jersey, USA) was used for capture. Fluorescence FITC images were acquired at 20× (512 × 512 pixels, 0.8 μm per pix.) objective magnification with an exposure time of 100 ms. After acquisition, ground truth counts of the FITC-positive MCF-7 cells were obtained from the images. It is important to note that WBCs were not tagged fluorescently and therefore do not show any signatures in these images.

### 5.8 Computational analysis

The automated analysis involving processing of raw holograms and training/testing of the ML models was performed on a desktop computer (Intel(R) Core(TM) i7-7700 CPU, 3.60 GHz, 16 GB RAM) using MATLAB (R2021b; MathWorks Inc., Natick, Massachusetts) software. The average processing speed per hologram is 5 seconds. For training the deep ResNet-50 Network, a GPU-enabled (NVIDIA GeForce GTX 1060, 6 GB) system (AMD Ryzen Threadripper 2990WX 32-Core Processor, 3.00 GHz, 32 GB RAM) was used. For the s-Net model, hyperparameters such as number of convolutional filters, filter size, number of epochs, minibatch size and learning rate of the optimizer *etc.* were independently tuned to achieve optimal generalized classification performance. In case of ResNet-50, optimization of the number of epochs, minibatch size and optimizer learning rate yielded the same values as those used for s-Net model training.

## Author contributions

AG and SAV conceptualized the study, AG designed and conducted the experiments, developed the analysis framework and generated all the data. AG and SAV wrote the paper. SAV and HSS provided feedback on the manuscript and supervised the project.

## Conflicts of interest

The authors do not have conflicts of interest to declare.

## Supplementary Material

RA-013-D2RA07972K-s001
