# Supplementary material for: Deep learning assisted holography microscopy for in-flow enumeration of tumor cells in blood
Source: RSC Adv. 2023 Feb 2;13(7):4222–35. doi: 10.1039/d2ra07972k (PMC9892890; doi:10.1039/d2ra07972k)
Supplement: RA-013-D2RA07972K-s001 [file RA-013-D2RA07972K-s001.pdf]

Electronic Supplementary Material (ESI) for RSC Advances.
This journal is © The Royal Society of Chemistry 2023

Deep Learning Assisted Holography Microscopy for In-Flow Enumeration of Tumor Cells in Blood

Anirudh Gangadhar [1], Hamed Sari-Sarraf[2] and Siva A. Vanapalli [1]

[1] Department of Chemical Engineering, Texas Tech University, Lubbock, TX 79409

[2] Department of Electrical and Computer Engineering, Texas Tech University, Lubbock, TX 79409

Supplementary Information

Table S1: s-Net versus other CNNs using reduced dataset ($N_{train} = 3500, N_{test} = 1500$)

CNN Network	Learnable parameters	Accuracy	Sensitivity	Specificity	Inference time (sec)
**s-Net (this work)**	**11,000**	**98.97%**	**97.68%**	**98.46%**	**4**
LeNet-5	61,310	97.09%	97.02%	97.16%	5
MobileNet-v2	3.5 million	97.54%	96.33%	98.76%	21
ResNet-50	25.5 million	97.55%	96.77%	98.34%	20

Table S2: MCF-7 ground truth and ML prediction counts for $C_{WBC} = 1000/mL$

**Target**	**Ground truth conc. (/mL)**					**ML-predicted conc. (/mL)**				
	**Donor 1**	**Donor 2**	**Donor 3**	**Mean**	**SD**	**Donor 1**	**Donor 2**	**Donor 3**	**Mean**	**SD**
0	0	0	0	0	0	1	1	2	1.33	0.58
10	12	7	9	9.33	2.52	7	1	5	4.33	3.06
100	83	115	94	97.33	16.26	30	37	41	36	5.57
1000	1040	925	917	960.67	68.82	183	243	225	217	30.79

Table S3: MCF-7 ground truth and ML prediction counts for $C_{WBC} = 5000/mL$

**Target**	**Ground truth conc. (/mL)**					**ML-predicted conc. (/mL)**				
	**Donor 1**	**Donor 2**	**Donor 3**	**Mean**	**SD**	**Donor 1**	**Donor 2**	**Donor 3**	**Mean**	**SD**
0	0	0	0	0	0	3	2	2	2.33	0.58
10	9	8	9	8.67	0.58	4	2	7	4.33	2.52
100	92	120	86	99.33	18.15	31	42	29	34	7
1000	945	1010	904	959.67	44.84	227	282	248	252.33	27.76

Table S4: SkOV3 ground truth and ML prediction counts for $C_{WBC} = 1000/mL$

**Target**	**Ground truth conc. (/mL)**					**ML-predicted conc. (/mL)**				
	**Donor 1**	**Donor 2**	**Donor 3**	**Mean**	**SD**	**Donor 1**	**Donor 2**	**Donor 3**	**Mean**	**SD**
0	0	0	0	0	0	5	2	0	2.33	2.52
10	12	8	9	9.67	2.08	5	10	15	10	5
100	87	102	91	93.33	7.77	31	24	54	36.33	15.70
1000	911	1033	979	974.33	61.13	240	181	301	240.67	60.00

Table S5: SkOV3 ground truth and ML prediction counts for $C_{WBC} = 5000/mL$

**Target**	**Ground truth conc. (/mL)**					**ML-predicted conc. (/mL)**				
	**Donor 1**	**Donor 2**	**Donor 3**	**Mean**	**SD**	**Donor 1**	**Donor 2**	**Donor 3**	**Mean**	**SD**
0	0	0	0	0	0	7	2	4	4.33	2.52
10	11	8	13	10.67	2.52	6	5	12	7.67	3.79
100	94	105	89	96	8.19	33	20	44	32.33	12.01
1000	945	987	1026	986	40.51	284	159	309	250.67	80.36

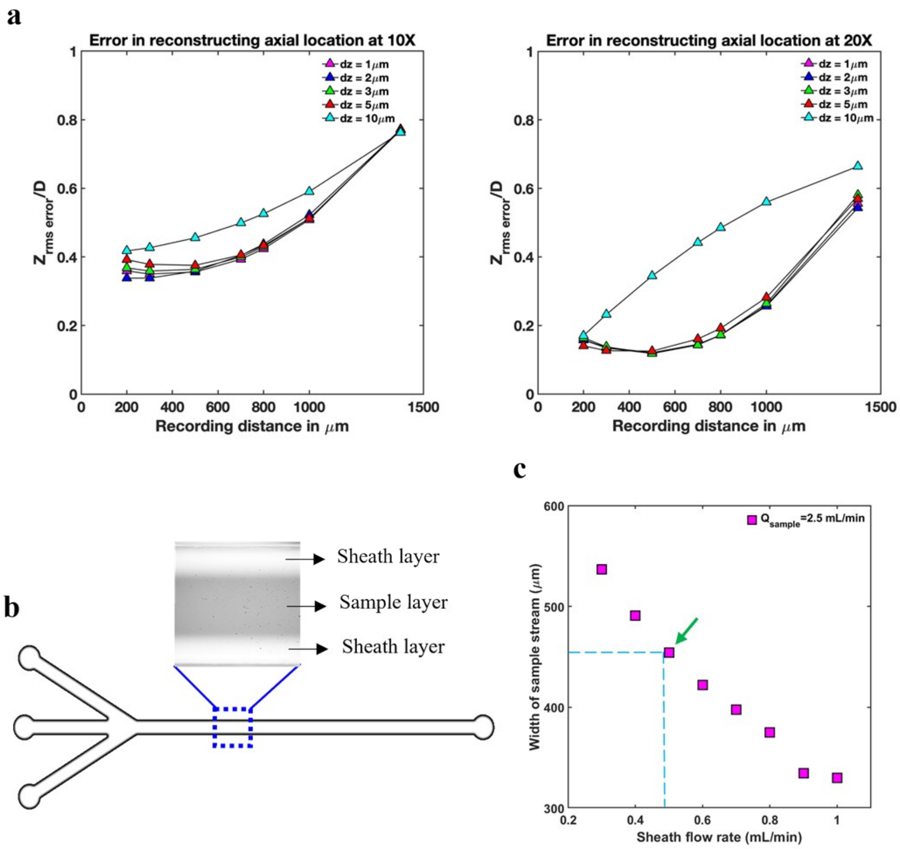

Figure S1. Optimization of critical DHM parameters and sheath flow conditions. (a) Normalized RMS error in reconstructing axial positions of 15 μm particles computed for various hologram recording distances tested, ranging from 200 to 1500 μm. Results are reported for 5 spacings between successive reconstruction planes: 1, 2, 3, 5 and 10 μm. Experiments are carried out under two objective magnifications, 10X and 20X; (b) Geometry of sheath microchannel with trifurcating inlets and a single outlet. Inset shows the sheath and sample regions in the image field of view (FOV). A black dye is used to visualize the separate regions and interfaces; (c) The operating sheath flow rate is optimized with respect to the width of the sample layer. Sample flow rate is fixed at 2.5 mL/min. The selected condition is highlighted by the green arrow.
